# Role of serum B-cell-activating factor and interleukin-17 as biomarkers in the classification of interstitial pneumonia with autoimmune features

**DOI:** 10.1515/biol-2022-0814

**Published:** 2024-02-08

**Authors:** Lihong Zhao, Li Liu, Yehua Liu, Hong Zheng, Ping Jiang

**Affiliations:** Department of Respiratory and Critical Care Medicine, Tianjin First Central Hospital, Tianjin, 300192, China; Department of Clinical Laboratory, Tianjin First Central Hospital, Tianjin, 300192, China; Department of Respiratory and Critical Care Medicine, Tianjin First Central Hospital, 24 Fukang Road, Nankai District, Tianjin, 300192, China

**Keywords:** interstitial lung disease, pneumonia autoimmune features, BAFF, interleukin-17, inflammatory mechanism

## Abstract

Interstitial pneumonia with autoimmune features (IPAF) is a type of interstitial lung disease (ILD) with immune features that do not meet the diagnostic criteria for specific connective tissue diseases (CTDs). This retrospective case–control study investigated the role of serum B-cell-activating factor of the tumor necrosis factor family (BAFF) and interleukin (IL)-17 as biomarkers for IPAF. The differences in serum BAFF, IL-17, and IL-10 were compared among patients with idiopathic pulmonary fibrosis (IPF), IPAF, ILD associated with CTD (CTD-ILD), and healthy controls. The patients were treatment naïve. The correlations of BAFF with IL-10, IL-17, and pulmonary function were analyzed. The classifiable value of BAFF for IPAF was examined. The results showed that the serum levels of BAFF and IL-17 in the IPAF and CTD-ILD groups were higher than in the IPF group. High BAFF levels and high predicted diffusion capacity of the lungs for carbon monoxide (DLCO) were independent predictive factors for IPAF vs IPF. In the IPAF and CTD-ILD groups, serum BAFF levels were negatively correlated with predicted values of forced vital capacity (FVC%) and diffusing capacity of the lungs for carbon monoxide (DLCO%) and positively correlated with serum IL-17 and IL-10 levels. The cutoff value of combined BAFF and IL-17 was 0.704, and the sensitivity and specificity for classifying IPAF were 78.9 and 95.7%, respectively. In conclusion, combining serum BAFF and IL-17 as a biomarker may have classifiable value in differentiating IPAF from other forms of ILD.

## 1 Introduction

Interstitial lung disease (ILD) is characterized by diffuse inflammation and/or fibrosis in the lung. Connective tissue diseases (CTDs) are inflammatory, immune-mediated disorders that can cause various pulmonary complications, especially different patterns of inflammation and fibrosis. Some ILDs associated with autoimmune features are not classifiable as CTDs. Some ILDs may be the only early manifestation of CTD. In 2015, the Connective Tissue Disease-Interstitial Lung Disease Working Group of the European Respiratory Society and the American Thoracic Society coined the term ‘interstitial pneumonia with autoimmune features’ (IPAF), established its classification criteria, and proposed the extrapulmonary clinical domains, serological domains, and morphological domains. IPAF can be classified by the presence of at least one feature from at least two of these domains [1]. Several studies have reported that the prognosis of ILD associated with CTD (CTD-ILD) is better than that of idiopathic pulmonary fibrosis (IPF) [2,3], and IPAF may be an early pulmonary manifestation of CTD. There are currently no management guidelines specific to IPAF.

IPAF often cannot be classified in time for many patients with ILD, especially those with nonspecific interstitial pneumonia (NSIP) with atypical extrapulmonary manifestations and negative or weakly positive serum autoantibody profiles, delaying treatment. Thus, an in-depth understanding of the inflammatory mechanism of IPAF and the identification of biomarkers to distinguish between IPAF and IPF is of great importance for the early diagnosis, treatment, and prognosis of the disease. Currently, there are limited studies on IPAF. Serum CXCL1, CXCL9, CXCL10, and CXCL11 are biomarkers of ILDs with immune features and can predict the therapeutic response of immunosuppressive agents [4,5]. Serum surfactant protein-A (SP-A), surfactant protein-D (SP-D), and Krebs von den Lungen-6 (KL-6) are inconsistent markers for evaluating alveolar epithelial cell injury and interstitial inflammation in the classification of IPAF. Some studies have reported that the above indicators have medium-high sensitivity and specificity for the classification of IPAF [6–8], but Kameda et al. found no difference in SP-A and SP-D levels among IPAF, IPF, and CTD-ILD [4].

B-cell-activating factor of the tumor necrosis factor (TNF) family (BAFF) is derived from monocytes, macrophages, dendritic cells, neutrophils, and activated T cells. BAFF is an important stimulating factor required for B-lymphocyte proliferation, differentiation, antibody secretion, and generation of effector T-cell-dependent immune response, which is closely related to various immune system diseases [9]. The BAFF levels in CTD-ILD are significantly higher than in chronic fibrotic ILD [10]. Given the immune features of IPAF, it is unclear whether the BAFF levels are as high as that of CTD and whether the immune-inflammatory mechanism of IPAF is similar to that of CTD.

In this study, the serum levels of BAFF, interleukin (IL)-10, and IL-17 were determined in patients with IPAF, CTD-ILD, and IPF, and the correlations among BAFF, IL-17, IL-10, lung function, and alveolar-arterial oxygen pressure difference (AaDO2) were analyzed. The significance of BAFF and IL-17 in the differential classification of IPAF and IPF was discussed.

## 2 Materials and methods

### 2.1 Study design and participants

It was a retrospective case–control study that included 19 patients with IPF, 23 patients with IPAF, and 23 patients with CTD-ILD, all hospitalized in the Department of Respiratory and Critical Care Medicine of Tianjin First Central Hospital between August 2019 and February 2021. The control group included 22 healthy subjects. All patients with IPF met the diagnostic criteria of the “Guidelines for the diagnosis and treatment of idiopathic pulmonary fibrosis” issued by the European Respiratory Society and the American Thoracic Society in 2011 [11]. Patients with IPAF met the classification criteria for IPAF [1] issued by the European Respiratory Society and the American Thoracic Society in 2015: extrapulmonary clinical domain, serological domain, and morphological domain classified by at least one feature from at least two of these domains. The diagnosis of CTD was in line with the diagnostic criteria for rheumatoid arthritis (2010) [12], the guidelines for diagnosis and treatment of idiopathic myositis (2017) [13], the guidelines for diagnosis and treatment of Sjogren’s syndrome (2016) [14], the Sharp criteria for mixed CTDs (1987) [15], and the guidelines for the diagnosis and treatment of systemic lupus erythematosus (2012) [16] issued by the American Rheumatism Society and European Rheumatism Society. All included patients were newly diagnosed and not yet treated by glucocorticoids, immunosuppressants, biological agents, or anti-fibrosis drugs. The included patients were older than 18 years and younger than 80 years. Patients with infection, malignant tumor, chronic heart, liver, or renal failure, ILD induced by drugs, dust, or radiation, sarcoidosis, allergic alveolitis, or those with missing lung function test data were excluded.

This study was reviewed and approved by the Ethics Committee of Tianjin First Central Hospital (Batch No. 2018N133KY). All subjects provided signed informed consent.


**Informed consent:** Informed consent has been obtained from all individuals included in this study.
**Ethical approval:** The research related to human use has been complied with all the relevant national regulations, institutional policies and in accordance with the tenets of the Helsinki Declaration, and has been approved by the Ethics Committee of Tianjin First Central Hospital (Batch No. 2018N133KY).

### 2.2 Collection of clinical information

The following patient data were recorded: sex, age, smoking history, arterial partial oxygen pressure (PaO_2_), alveolar-arterial oxygen pressure difference (AaDO_2_), and chest high-resolution computed tomography (HRCT). The HRCT manifestations were evaluated by two respiratory physicians and two radiologists with more than 10 years of experience and classified into NSIP, usual interstitial pneumonia (UIP), and others (except NSIP and UIP, such as organizing pneumonia, lymphocytic interstitial pneumonia, etc.).

### 2.3 Pulmonary function tests (PFTs)

PFTs were performed using the pulmonary function instrument (Jaeger, Germany) as per the ATS and ERS guidelines. The tests measured forced vital capacity (FVC) and the diffusion capacity of the lungs for carbon monoxide (DLCO).

### 2.4 Sample processing and measurement of serum markers

Fasting venous blood (4 ml) was drawn into an EDTA anticoagulant tube and centrifuged at 3,000 rpm for 10 min, with a radius of 6 cm. The supernatant was transferred to a clean EP tube and stored at −80°C. Serum BAFF, IL-10, and IL-17 levels were determined by enzyme-linked immunosorbent assay (ELISA). The absorbance was measured at 450 nm using an automatic microplate analyzer. The standard curve was drawn, and the sample concentration was calculated based on the absorbance value. The ELISA kits for IL-10, IL-17, and BAFF were purchased from Shanghai Fanke Biological Technology Co., Ltd.

### 2.5 Statistical analysis

Data were analyzed using SPSS 25.0 (SPSS, Inc., Chicago, IL, USA). Figures were drawn using Prism 9.0 (GraphPad Inc., La Jolla, CA, USA). The Kolmogorov–Smirnov test was used to verify the normal distribution. Categorical data were expressed as *n* (%), and continuous data were expressed as mean ± standard deviation. In the univariable analysis, categorical variables were compared using the chi-square test, which includes Pearson’s chi-square test and Fisher’s exact probability method, to assess inter-group differences. Continuous variables, on the other hand, were subjected to analysis of variance for inter-group comparisons, followed by post hoc analysis for pairwise comparisons. Pearson’s analysis was used for correlation. Univariable regression analyses were performed to examine the factors associated with IPF vs non-IPF. A multivariable regression analysis was performed to examine the association of variables with non-IPF after adjustment for age, sex, and smoking. Univariable and multiple (stepwise) logistic regression analyses were conducted to investigate the factors associated with IPAF vs IPF, IPAF vs CTD-ILD, and CTD-ILD vs IPF. The receiver operating characteristic (ROC) curve was drawn to calculate the optimal cutoff value, corresponding sensitivity and specificity based on Youden’s index, and the classifiable value of serum BAFF and IL-17 in identifying IPAF and IPF. *P* < 0.05 indicated a significant difference.

## 3 Results

### 3.1 Characteristics of the patients

This study included 23 patients in the IPAF group, 23 in the CTD-ILD group, 19 in the IPF group, and 22 healthy individuals. There were 4 males (17.4%) and 19 females (82.6%), aged 61.0 ± 10.6 years in the IPAF group, 5 males (21.7%) and 18 females (78.3%), aged 63.7 ± 12.5 years in the CTD-ILD group, 14 males (73.7%) and 5 females (26.3%), aged 67.6 ± 5.4 years in the IPF group, and 10 males (45.5%) and 12 females (54.5%), aged 61.7 ± 4.1 years in the control group. There were no significant differences in age among the four groups. Most patients in the IPAF and CTD-ILD groups were female (82.6 vs 78.3 vs 26.3%, *p* = 0.001). There were 3 (13.0%) smokers in the IPAF group, 6 (26.1%) in the CTD-ILD group, and 10 (52.6%) in the IPF group, with significantly more smokers in the IPF group (*p* = 0.022). The predicted DLCO%, AaDO_2_, serum BAFF, IL-17, and IL-10 levels were statistically different among the four groups (all *p* < 0.05), as shown in Table 1.

**Table 1:** Comparison of the clinical characteristics of the IPAF, CTD-ILD, and IPF groups with the healthy controls

Variable	IPAF (*n* = 23)	CTD-ILD (*n* = 23)	IPF (*n* = 19)	HC (*n* = 22)	*p*
Age, years	61.0 ± 10.6	63.7 ± 12.5	67.6 ± 5.4	61.7 ± 4.1	0.093
Female, *n* (%)	19 (82.6)	18 (78.3)	5 (26.3)	12 (54.5)	0.001
Smoker, *n* (%)	3 (13.0)	6 (26.1)	10 (52.6)	8 (36.4)	0.022
HRCT type, *n* (%)					
NSIP	16 (69.6)	12 (52.2)	9 (47.4)		0.119
UIP	4 (17.4)	8 (34.8)	10 (52.6)		0.089
Other	3 (13.0)	3 (13.0)	0 (0)		0.123
Predicted FVC%	71.3 ± 17.7	73.1 ± 15.7	63.7 ± 15.3		0.158
Predicted DLCO%	46.7 ± 16.8	51.8 ± 15.8	30.1 ± 14.5		<0.001
AaDO_2_ (mmHg)	31.6 ± 11.5	24.9 ± 14.0	38.3 ± 12.7		0.005
BAFF (ng/ml)	1.9 ± 1.2	2.0 ± 1.4	1.0 ± 0.2	0.7 ± 0.2	<0.001
IL-17 (pg/ml)	8.5 ± 4.6	8.8 ± 6.4	5.4 ± 0.5	5.0 ± 1.7	0.002
IL-10 (pg/ml)	182.1 ± 92.7	177.4 ± 138.5	126.2 ± 11.5	99.1 ± 63.3	0.007

### 3.2 Comparison of serum BAFF, IL-17, and IL-10 among the IPAF, CTD-ILD, IPF, and healthy control groups

**Figure 1:**
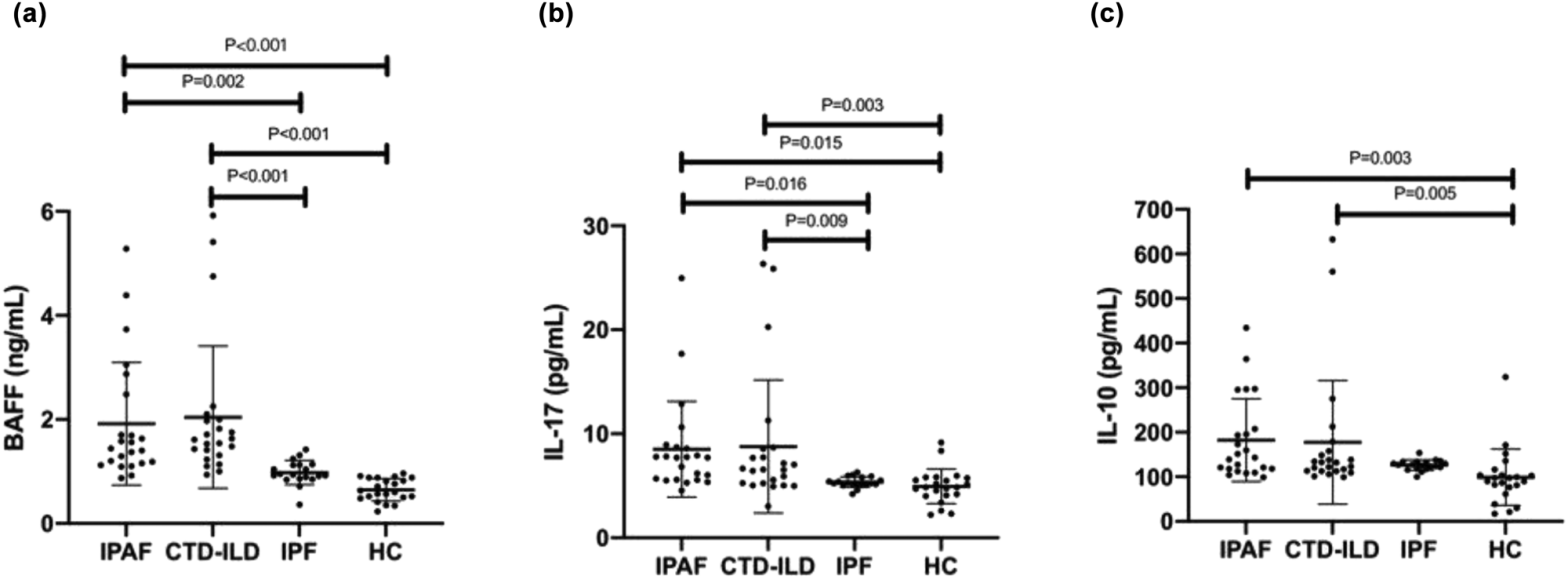
Distribution of serum (a) BAFF, (b) IL-17, and (c) IL-10 in the IPAF (*n* = 23), CTD-ILD (*n* = 23), IPF (*n* = 19), and HC (*n* = 22) groups. A pairwise comparison of variables between the groups was performed using post hoc analysis. BAFF: B-cell-activating factor of the TNF family; IL-17: interleukin-17; IL-10: interleukin-10; IPAF: interstitial pneumonia with autoimmune features; CTD-ILD: interstitial lung disease associated with connective tissue disease; IPF: idiopathic pulmonary fibrosis; HC: healthy control.

The serum levels of BAFF, IL-17, and IL-10 in the IPAF and CTD-ILD groups were significantly higher than in the healthy control group (*p* < 0.001, *p* = 0.005, *p* = 0.003; *p* < 0.001, *p* = 0.003, *p* = 0.005). The levels in the IPF group were not significantly different from the healthy control group. Compared with the IPF group, the serum BAFF and IL-17 levels in the IPAF and CTD-ILD groups were significantly higher (*p* = 0.002, *p* < 0.001; *p* = 0.016, *p* = 0.009). There were no significant differences between the CTD-ILD group and the IPAF group. There were no significant differences in IL-10 levels in the IPAF and CTD-ILD groups compared with the IPF group. There were no significant differences between the IPAF and CTD-ILD groups, as shown in Figure 1. After adjusting for age, sex, and smoking, BAFF levels were independently associated with non-IPF (adjusted odds ratio [aOR] = 0.001, 95% confidence interval [CI]: 0–0.076, *p* = 0.008), while IL-17 levels were not (aOR = 0.51, 95% CI: 0.15–1.35, *p* = 0.21) (Table 2). In the regression analysis for IPAF vs IPF, high predicted DLCO, BAFF, and IL-17 were risk factors for IPAF compared to IPF. Based on stepwise multiple logistic regression analysis, high BAFF levels and high predicted DLCO were independent predictive factors for IPAF vs IPF (aOR = 1.23, 95% CI: 1.11–1.38, *p* < 0.001; aOR = 1.01, 95% CI: 1.00–1.02, *p* < 0.001) (Table 3). There were no statistically significant differences in the characteristics between the IPAF and CTD-ILD groups. High BAFF levels and high predicted DLCO are independent predictive factors for CTD-ILD vs IPF (aOR = 0.87, 95% CI: 0.79–0.95, *p* < 0.001; aOR = 0.99, 95% CI: 0.98–0.99, *p* < 0.001).

**Table 2:** Comparison of the clinical characteristics between the IPF and non-IPF groups using univariable and multivariable logistic regression analyses

	Non-IPF (*n* = 46)	IPF (*n* = 19)	OR 95% CI	*p*	aOR 95% CI	*p*
Variable						
Age, years	62.3 (11.5)	67.6 (5.40)	1.07 [0.99;1.14]	0.07	1.05 [0.96, 1.21]	0.352
Sex, %						
Male	9 (19.6%)	14 (73.7%)	Ref.	Ref.	Ref.	Ref.
Female	37 (80.4%)	5 (26.3%)	0.09 [0.02;0.31]	<0.001	0.1 [0.01, 0.69]	0.028
Smoking, %						
No	37 (80.4%)	9 (47.4%)	Ref.	Ref.	Ref.	Ref.
Yes	9 (19.6%)	10 (52.6%)	4.42 [1.39;14.8]	0.012	1.22 [0.12, 11.95]	0.861
HRCT type, %						
NSIP	28 (60.9%)	9 (47.4%)	Ref.	Ref.		
UIP	12 (26.1%)	10 (52.6%)	—	—		
Other	6 (13.0%)	0 (0.00%)	—	—		
Predicted FVC%	72.2 (16.5)	63.7 (15.3)	0.97 [0.94;1.00]	0.064		
Predicted DLCO%	49.2 (16.3)	30.1 (14.5)	0.91 [0.87;0.96]	<0.001		
AaDO_2_ (mmHg)	28.2 (13.1)	38.3 (12.7)	1.07 [1.02;1.13]	0.011		
BAFF (ng/ml)	1.98 (1.26)	0.98 (0.23)	0.00 [0.00;0.06]	0.001	0.001 (0, 0.076)	0.008
IL-17 (pg/ml)	8.64 (5.51)	5.35 (0.50)	0.37 [0.18;0.74]	0.005	0.51 (0.15, 1.35)	0.21
IL-10 (pg/ml)	180 (117)	126 (11.5)	0.98 [0.96;1.00]	0.090		

IPF: idiopathic pulmonary fibrosis; OR: odds ratio; CI: confidence interval; aOR: adjusted odds ratio; HRCT: high-resolution computed tomography; NSIP: nonspecific interstitial pneumonia; UIP: usual interstitial pneumonia; FVC: forced vital capacity; DLCO: diffusion capacity of the lungs for carbon monoxide; AaDO_2_: alveolar-arterial oxygen pressure difference; BAFF: B-cell-activating factor of the TNF family; IL: interleukin.

**Table 3:** Comparison of the clinical characteristics between the IPAF and IPF groups using univariable and stepwise multivariable logistic regression analyses

	Non-IPF (*n* = 46)	IPF (*n* = 19)	OR 95% CI	*p*	aOR 95% CI	*p*
Variable						
Age, years	61.0 (10.6)	67.6 (5.4)	0.9 [0.82; 0.99]	0.03	0.99 [0.98, 1.00]	0.19
Sex, %						
Male	4 (17.4%)	14 (73.7%)	Ref.	Ref.	Ref.	Ref.
Female	19 (82.6%)	5 (26.3%)	13.3 [3.01; 58.72]	<0.001		
Smoking, %						
No	20 (87.0%)	9 (47.4%)	Ref.	Ref.	Ref.	Ref.
Yes	3 (13.0%)	10 (52.6%)	0.14 [0.03; 0.65]	0.01	0.74 [0.58, 0.94]	0.01
HRCT type, %						
NSIP	16 (69.6%)	9 (47.4%)	Ref.	Ref.	Ref.	Ref.
UIP	4 (17.4%)	10 (52.6%)	0.22 [0.05; 0.93]	0.04	0.94 [0.73; 1.23]	0.66
Other	3 (13.0%)	0 (0.00%)	—	—		
Predicted FVC%	71.3 (17.7)	63.7 (15.3)	1.03 [0.99; 1.07]	0.15		
Predicted DLCO%	46.7 (16.8)	30.1 (14.5)	1.07 [1.02; 1.13]	0.01	1.01[1.00; 1.02]	<0.001
AaDO_2_ (mmHg)	31.6 (11.5)	38.3 (12.7)	0.95 [0.9; 1.01]	0.09		
BAFF (ng/ml)	1.90 (1.20)	0.98 (0.23)	386.16 [6.71; 22234.28]	<0.001	1.23 (1.11, 1.38)	<0.001
IL-17 (pg/ml)	8.50 (4.60)	5.35 (0.50)	5.09 [1.54; 16.78]	0.01		
IL-10 (pg/ml)	182.1(92.7)	126.2 (11.5)	1.02 [1;1.05]	0.06		

IPAF: interstitial pneumonia with autoimmune features; IPF: idiopathic pulmonary fibrosis; OR: odds ratio; CI: confidence interval; aOR: adjusted odds ratio; HRCT: high-resolution computed tomography; NSIP: nonspecific interstitial pneumonia; UIP: usual interstitial pneumonia; FVC: forced vital capacity; DLCO: diffusion capacity of the lungs for carbon monoxide; AaDO2: alveolar-arterial oxygen pressure difference; BAFF: B-cell-activating factor of the TNF family; IL: interleukin.

### 3.3 Correlation of BAFF with lung function, AaDO_2_, IL-10, and IL-17

**Figure 2:**
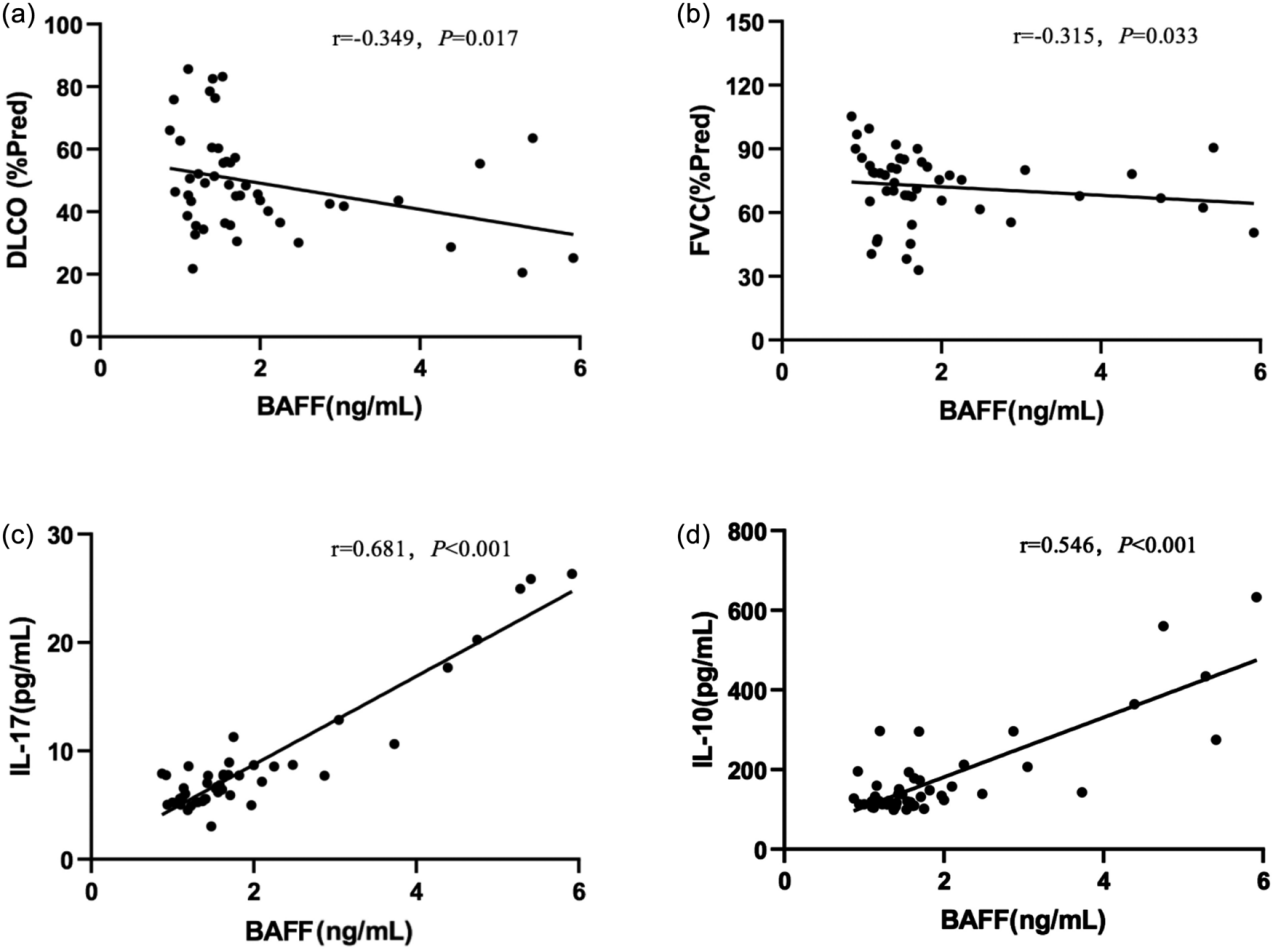
Correlation analysis of serum BAFF with (a and b) PFT parameters, (c) IL-17, and (d) IL-10 in the IPAF and CTD-ILD groups. BAFF: B-cell-activating factor of the TNF family; IL-17: interleukin-17; IL-10: interleukin-10; FVC: forced vital capacity; DLCO: diffusing capacity for carbon monoxide; IPAF: interstitial pneumonia with autoimmune features; CTD-ILD: interstitial lung disease associated with connective tissue disease.

This study analyzed the correlations of serum BAFF with predicted values of FVC% and DLCO%, AaDO_2_, IL-10, and IL-17 in the IPAF and CTD-ILD groups. The serum BAFF levels were negatively correlated with the predicted DLCO% and FVC% (*r* = −0.349, *p* = 0.017; *r* = −0.315, *p* = 0.033), and positively correlated with serum IL-17 and IL-10 levels (*r* = 0.681, *p* < 0.001; *r* = 0.546, *p* < 0.001), as shown in Figure 2. There were no significant correlations between serum BAFF levels and AaDO_2_.

### 3.4 Classifiable value of serum BAFF and IL-17 levels for IPAF

**Figure 3:**
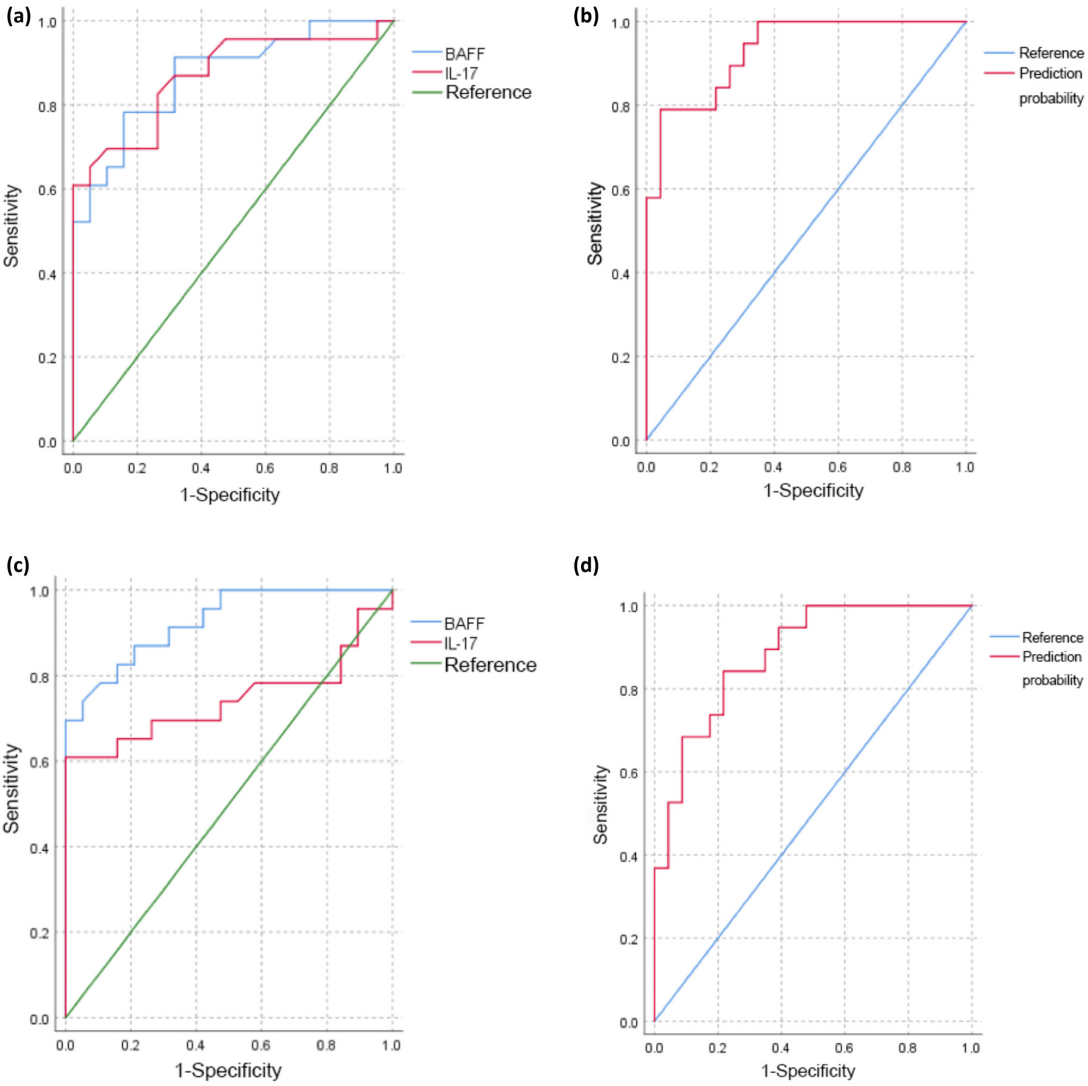
(a and b) ROC curve analysis of different serum BAFF and IL-17 levels between patients with IPAF and IPF. (c and d) ROC curve analysis of different serum BAFF and IL-17 levels between patients with CTD-ILD and IPF. BAFF: B-cell-activating factor of the TNF family; IL-17: interleukin-17.

We used an ROC curve analysis to evaluate the sensitivity and specificity of serum BAFF and IL-17 levels as biomarkers for the differential classification of IPAF from IPF. Based on the area under the ROC curve (AUC), when the cutoff value for BAFF was 1.150 ng/ml, the sensitivity and specificity were 78.3 and 84.2%, respectively (Youden’s index: 0.625, AUC = 0.871, 95% CI = 0.765–0.976). When the cutoff value for IL-17 was 6.565 pg/ml, the sensitivity and specificity were 60.9 and 100%, respectively (Youden’s index: 0.609, AUC = 0.868, 95% CI = 0.759–0.978). When the cutoff value of BAFF and IL-17 combination was 0.704, the sensitivity and specificity were 78.9 and 95.7%, respectively (Youden’s index: 0.746, AUC = 0.931, 95% CI = 0.86–1.00) (Figure 3a and b). When differentiating CTD-ILD from IPF, using a cutoff value of 0.596, BAFF and IL-17 combination achieved 84.2% sensitivity and 78.3% specificity (Youden’s index: 0.625, AUC = 0.883, 95% CI = 0.786–0.981), while BAFF alone achieved 69.6% sensitivity and 100% specificity using a cutoff of 1.427 (Youden’s index: 0.696, AUC = 0.926, 95% CI = 0.852–1.000) and IL-17 alone achieved 60.9% sensitivity and 100% specificity using a cutoff of 6.37 (Youden’s index: 0.609, AUC = 0.743, 95% CI = 0.584–0.901) (Figure 3c and d).

## 4 Discussion

The serum levels of BAFF, IL-17, and IL-10 in the ILD groups and the control group were determined in this study. The results showed that the serum BAFF and IL-17 in the IPAF and CTD-ILD groups were significantly higher than in the IPF and control groups. High serum BAFF levels and high predicted DLCO were independent predictive factors for IPAF vs IPF. There were no significant differences in BAFF and IL-17 between CTD-ILD and IPAF groups. The serum BAFF levels were negatively correlated with the predicted FVC% and DLCO%. The sensitivity and specificity of the serum BAFF and IL-17 combination as a biomarker for the differential classification of IPAF from other types of ILD were high. Despite the absence of guidelines for IPAF management, identifying biomarkers of IPAF is of great importance for determining the autoimmune cause of ILD. Both BAFF and IL-17 are inflammatory markers, indicating that CTD-ILD and IPAF might share the same or a similar inflammatory response. Therefore, when BAFF and IL-17 were elevated in ILD, it suggested some autoimmune factors, as previously suggested [17,18].

BAFF is an important stimulator of B-lymphocyte proliferation, differentiation, antibody secretion, and generation of effector T-cell-dependent immune response. Autoantibodies are produced by BAFF-activated B lymphocytes and released into the blood circulation. The expression of BAFF is significantly increased in systemic lupus erythematosus, rheumatoid arthritis, Sjogren’s syndrome, systemic sclerosis, and dermatomyositis and is closely related to disease activity, autoantibody production, and titer [19–23]. Serum BAFF levels are significantly increased in the early stages of polymyositis and rheumatoid arthritis, suggesting that BAFF is involved in the early onset of the disease [19,22]. Blocking BAFF activity decreases the severity of autoimmune diseases such as chronic colitis [24]. Belimumab is an anti-BAFF therapy under exploration for managing CTD-ILD [25]. Autoantibody reduction therapy has been shown to improve the lung function of patients with IPF [26]. A previous study showed that BAFF levels combined with ultrasound B lines could effectively differentiate among different types of IPFs, including IPAF [18]. This study showed that the serum BAFF levels of the patients in the IPAF and CTD-ILD groups were higher than in the IPF group, and the BAFF levels in the CTD-ILD group were higher than in the IPAF group. It indicates that IPAF is different from IPF and may be consistent with the inflammatory nature of CTD-ILD. BAFF was also independently associated with IPAF after adjustment for age, sex, and smoking. IPAF can occur in the early stage of CTD but without the inflammation intensity typical of CTD. Hence, biomarkers for the early classification of IPAF could be useful.

BAFF levels in dermatomyositis and mixed CTD patients with interstitial pneumonia were significantly higher than in patients without [22,27]. In addition, serum BAFF levels in the CTD-ILD group were significantly higher than in patients with chronic fibrous interstitial pneumonia and healthy controls and were negatively correlated with the predicted FVC% in the CTD-ILD group. Immunohistochemical analysis of lung specimens of CTD-ILD patients revealed that BAFF was strongly positive in the alveolar wall, peripheral airway, and lymphatic follicles [10]. In addition, BAFF is higher in UIP induced by autoimmune diseases than in IPF and negatively correlated with lung function [28]. As supported by the above studies, the present study showed that the BAFF levels were negatively correlated with predicted FVC% and DLCO%. Thus, the BAFF levels might be used to assess the extent of autoimmune inflammation in IPAF.

In addition to the involvement of BAFF in the differentiation of CTDs as an effector inflammatory cytokine secreted by T lymphocytes, ILs also play important roles in this process. IL-17, an effector cytokine secreted by Th17 cells, is a landmark pro-inflammatory factor that can induce a cascade amplification effect of other pro-inflammatory factors and chemokines and further trigger inflammatory responses, playing an important role in the pathogenesis of various CTDs. BAFF upregulates the expression of CD28/B7 and CD40/CD154 by binding to BAFF receptors and promotes the interaction between T and B lymphocytes, resulting in the generation of IL-17 and autoantibodies [29]. Patients with positive anti-tRNA synthase antibodies and ILD develop lung disease progression and show continuously elevated cytokines secreted by Th17 [30]. In this study, the levels of serum IL-17 in the IPAF and CTD-ILD groups were higher than in the IPF group, supporting the similarity between IPAF and CTD-ILD inflammation. Still, IL-17 levels were not independently associated with non-IPF after adjustment for age, sex, and smoking.

Recently, clinicians have been looking for effective biomarkers to distinguish between IPAF and IPF. KL-6, SP-D, SP-A, CXCL1, CXCL9, CXCL10, and CXCL11 have been reported, but the results are not consistent, and the proposed biomarkers cannot effectively distinguish between IPF, IPAF, and CTD-ILD [4–8,31]. As a superior regulator of serum autoantibodies, BAFF reflects the changes in autoimmune inflammation earlier and is more sensitive than autoantibodies. IL-17, as a signature pro-inflammatory factor of autoimmune inflammation, can be used as an effective biomarker to distinguish IPAF from IPF. Better sensitivity and specificity can be achieved by combining BAFF and IL-17. NSIP is the most common tissue type of IPAF and CTD-ILD, and about 10–52% of patients with idiopathic NSIP are eventually diagnosed with a specific type of CTD during follow-up [32,33]. Thus, for patients with ILD, especially patients with NSIP (accounting for 69.6% of the IPAF group in this study), the combination of BAFF and IL-17 can help to distinguish IPAF from other types of ILDs. Whether treatment with glucocorticoids and immunosuppressors could also avoid or delay the damage to other organs remains to be examined.

This study had some limitations. It was a single-center retrospective study with a small sample size, and there may be some selection bias. The cross-sectional nature of the study and the lack of longitudinal assessments prevented the determination of cause-to-effect relationships and the predictive value of BAFF and IL-17 for IPAF. It is necessary to conduct multicenter prospective studies and increase the sample size in the future. Besides, histopathological verification was not conducted in this study, which needs further confirmation.

In conclusion, the combination of BAFF and IL-17 is a sensitive biomarker for differentiating IPAF and IPF, which has great clinical significance. IPAF and CTD-ILD differ from IPF, and both have similar inflammatory characteristics. IPAF may be the early stage of CTD, and the treatment should focus on CTD. Early intervention could avoid multiple organ injuries, affecting patients’ quality of life and prognosis.
